# A Comprehensive Review on Biofilms in Otorhinolaryngology: Understanding the Pathogenesis, Diagnosis, and Treatment Strategies

**DOI:** 10.7759/cureus.57634

**Published:** 2024-04-04

**Authors:** Ayushi Ghosh Moulic, Prasad Deshmukh, Sagar S Gaurkar

**Affiliations:** 1 Otorhinolaryngology, Jawaharlal Nehru Medical College, Datta Meghe Institute of Higher Education & Research, Wardha, IND

**Keywords:** chronic rhinosinusitis, treatment, diagnosis, pathogenesis, otorhinolaryngology, biofilms

## Abstract

Biofilms, structured communities of microorganisms encased in a self-produced matrix, pose significant challenges in otorhinolaryngology due to their role in chronic and recurrent infections affecting the ear, nose, and throat (ENT) region. This review provides an overview of biofilms, emphasizing their formation, pathogenesis, diagnosis, and treatment strategies in otorhinolaryngological disorders. Biofilms are pivotal in chronic rhinosinusitis (CRS), otitis media, laryngopharyngeal reflux (LPR), and tonsillitis, contributing to treatment resistance and disease recurrence. Current diagnostic techniques, including imaging modalities, microbiological cultures, and molecular techniques, are discussed, alongside emerging technologies. Treatment strategies, ranging from conventional antibiotics to alternative therapies, such as biofilm disruptors, phage therapy, and immunomodulation, are evaluated in terms of their efficacy and potential clinical applications. The review underscores the significance of understanding biofilms in otorhinolaryngology and highlights the need for tailored approaches to diagnosis and management to improve patient outcomes.

## Introduction and background

Biofilms are structured communities of microorganisms that adhere to surfaces and are encapsulated within a self-produced matrix of extracellular polymeric substances (EPS) [1]. Unlike planktonic (free-floating) microbes, biofilm-associated microorganisms exhibit increased resistance to antimicrobial agents and host defenses, making them challenging to eradicate. These complex microbial communities are significant in various infectious diseases across medical fields, including otorhinolaryngology [2].

In otorhinolaryngology, biofilms are implicated in the pathogenesis of numerous chronic and recurrent infections affecting the ear, nose, and throat (ENT) region [3]. Conditions such as chronic rhinosinusitis (CRS), otitis media, laryngopharyngeal reflux (LPR), and tonsillitis are often associated with biofilm formation [4]. The ability of biofilms to persist within the intricate anatomy of the ENT system contributes to treatment resistance and disease recurrence, posing significant challenges for clinicians [5].

This review aims to provide a comprehensive understanding of biofilms in otorhinolaryngology, focusing on their pathogenesis, diagnosis, and treatment strategies. By exploring the mechanisms underlying biofilm formation and their implications in various ENT disorders, this review aims to shed light on the clinical significance of biofilms and the challenges they pose in patient management. Additionally, the review discusses current diagnostic techniques and therapeutic approaches while highlighting emerging research directions and potential future interventions. Ultimately, this review seeks to contribute to advancing knowledge in the field of otorhinolaryngology and improve patient outcomes by addressing the complex role of biofilms in ENT infections.

## Review

Biofilm formation in otorhinolaryngology

Mechanisms of Biofilm Formation

Forming biofilms involves a series of intricate steps that bacteria undergo to establish these complex microbial communities. Initially, bacteria adhere to a surface using surface proteins, facilitating cell adhesion and resulting in irreversible attachment [6]. Subsequently, bacteria proliferate and produce extracellular polymeric substances (EPS), which play a vital role in shaping the three-dimensional structure of the biofilm and maintaining its integrity [6]. The EPS matrix offers physical protection to the clustered biofilm cells, enhancing their resistance to external factors, such as antibiotics and host immune responses [7]. Additionally, biofilm formation progresses through four primary stages: cellular adhesion, microcolony formation, biofilm maturation, and dispersion [6]. During the maturation phase, biofilms develop a protective extracellular matrix (ECM) that shields them from antibodies, phagocytosis, antibiotic penetration, and complement binding, rendering them considerably more resistant to antibiotic treatment than planktonic bacteria [6]. Eventually, biofilms undergo dispersion, releasing bacteria into the surrounding environment, thus perpetuating their presence and potential for causing infections [6]. Understanding these mechanisms is imperative for developing effective strategies to control and manage biofilm-related challenges across various medical domains, including otorhinolaryngology.

Host-Microbe Interactions

Host-microbe interactions are pivotal in shaping human health and disease across various conditions, ranging from chronic illnesses such as rhinosinusitis to prevalent pediatric ailments like otitis media (OM). These interactions entail a complex interplay between microorganisms and the human host, influencing disease pathogenesis and treatment outcomes [8,9]. Understanding these intricate dynamics is paramount for unraveling the underlying mechanisms of diseases and devising effective therapeutic interventions. The research underscores the significance of host-microbe interactions in various medical contexts. For instance, investigations have elucidated how microbial communities influence the colonization of pathogens in conditions such as OM, where specific bacteria contribute to disease susceptibility [8]. Similarly, biofilms observed in CRS serve as a valuable model for studying microbe-microbe and host-microbe interactions, providing insights into the altered physiological conditions associated with these disorders [9]. The field of host-microbe interactions encompasses diverse research areas, including genetics, microbiome studies, and the development of novel antimicrobial agents. By delving into the molecular mechanisms governing these relationships, researchers endeavor to enhance our understanding of disease pathogenesis and immune responses [10]. This deeper comprehension holds the promise of guiding the development of innovative treatment modalities and preventive strategies to mitigate microbial dysbiosis's impact and foster improved health outcomes.

Factors Influencing Biofilm Formation in the Ear, Nose, and Throat (ENT) Region

In the otorhinolaryngology (ENT) field, several factors contribute to the formation and persistence of biofilms, particularly on medical devices utilized within this region. First, surface characteristics of ENT medical devices, including implants for the trachea, cochlear implants, and voice prosthetics, significantly influence biofilm development. Techniques such as surface modifications and functionalization are pivotal in mitigating biofilm formation while promoting tissue regeneration [11]. Microbial colonization represents another critical aspect of biofilm formation in the ENT region. Microbes adhere to surfaces within the ENT system, initiating biofilm formation. This process involves the reversible attachment of bacteria followed by the elaboration of a glycocalyx, which leads to irreversible adhesion and subsequent biofilm growth. Such mechanisms are especially pertinent in chronic conditions such as OM with effusion (COME) [12].

**Figure 1:**
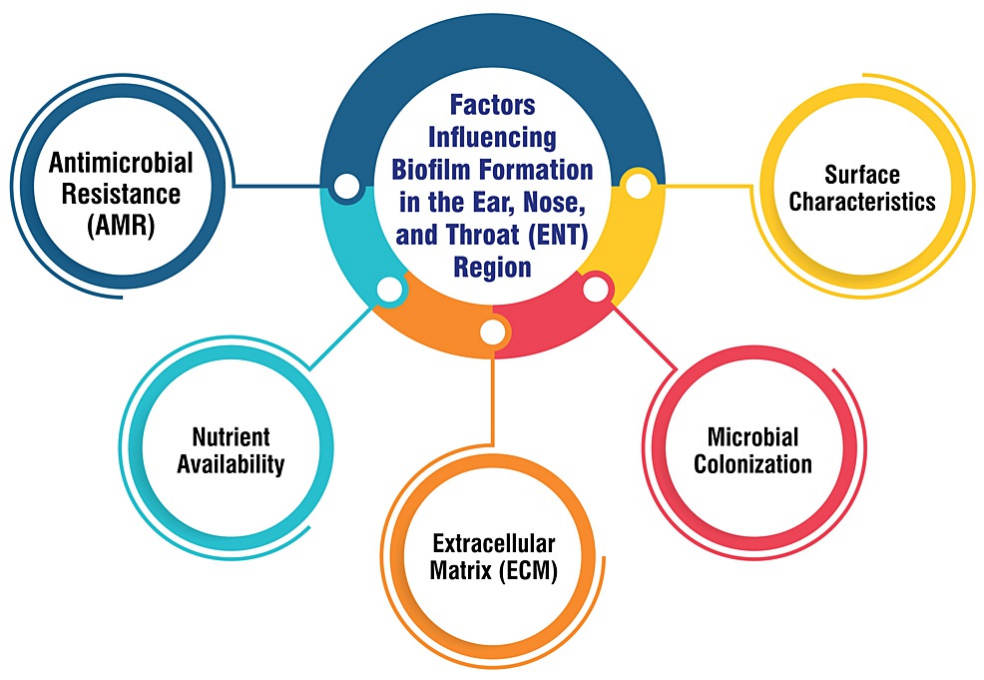
Factors influencing biofilm formation in the ENT Corresponding author Dr. Ayushi Ghosh Moulic created this figure.

Within biofilms, the ECM is crucial in enhancing water retention, carbon storage, antibiotic resistance, and shaping biofilms' architecture. Components such as extracellular DNA, proteins, and polysaccharides contribute to the structural integrity of biofilms [13]. Nutrient availability within the ECM influences biofilm development in the ENT region. The presence of nutrients such as sugars affects the acidity of the microenvironment and facilitates changes that support bacterial growth and survival, thereby influencing biofilm formation [14]. Moreover, biofilms exhibit antimicrobial resistance (AMR), posing significant challenges for treatment strategies in otorhinolaryngology. The inherent self-protective nature of biofilms contributes to chronic infections and device-related issues. Thus, understanding and addressing AMR mechanisms are imperative in managing biofilm-related conditions effectively within ENT [15,16]. Considering these factors influencing biofilm formation in the ENT region, healthcare professionals can effectively devise targeted strategies to prevent, diagnose, and treat biofilm-related conditions within otorhinolaryngology. By addressing surface characteristics, microbial colonization, ECM composition, nutrient availability, and AMR, clinicians can optimize patient care and enhance treatment outcomes in ENT-related biofilm infections. Factors influencing biofilm formation in the ENT Region are shown in Figure 1.

Pathogenesis of biofilms in otorhinolaryngology

Role of Biofilms in CRS

Biofilms substantially impact CRS by influencing the condition's pathophysiology and therapeutic approaches. The research underscores the pivotal role of biofilms in chronic infections, including CRS, as studies have revealed their presence in a significant proportion of CRS cases [17,18]. These biofilms complicate infection management by fostering antibiotic resistance and triggering inflammatory responses, exacerbating clinical outcomes and disease severity [17,18]. Notably, in CRS patients, biofilms have been linked to elevated bacterial loads, heightened symptom scores, and increased postoperative symptoms, underscoring their contribution to disease progression and resistance to treatment [18]. Therapeutic strategies targeting biofilms aim to counteract biofilm microbes, disperse existing biofilms, or disrupt quorum sensing to enhance treatment efficacy in CRS [18]. Recognizing the functional significance of biofilms and their role in CRS is imperative for reshaping treatment approaches and devising more efficacious therapies that specifically target these resilient microbial communities.

Biofilms and OM

Biofilms play a pivotal role in OM, particularly chronic OM, by contributing to the persistence and resistance of bacterial infections within the middle ear. These microbial communities pose significant challenges as they are difficult to cultivate in laboratory settings and exhibit substantial resistance to antimicrobial agents, complicating the diagnosis and treatment of chronic OM [19,20]. Research has elucidated that bacterial pathogens linked to OM may reside intracellularly or within biofilms, underscoring the importance of comprehending these mechanisms for effective disease management [19]. Moreover, studies have identified biofilm-forming bacteria in the middle-ear mucosa of individuals with chronic OM, further underscoring the significance of biofilms in this condition [21]. Furthermore, recent investigations have concentrated on biofilm formation in children with complex OM, shedding light on the implications of biofilm-forming bacteria in this vulnerable population in the post-heptavalent pneumococcal conjugate vaccination era [22].

Biofilms in Laryngopharyngeal Reflux (LPR) and Laryngitis

Biofilms play a significant role in LPR and laryngitis, contributing to chronic infections and inflammation in the larynx and pharynx. In LPR, biofilms can form on the laryngeal tissues due to exposure to refluxed microorganisms and irritants, resulting in prolonged diffuse laryngeal inflammation often diagnosed as chronic laryngitis [23]. The biofilms in the larynx can exacerbate the inflammatory response and contribute to the persistence of laryngitis symptoms, impacting vocal quality and overall laryngeal health. Moreover, biofilms associated with chronic laryngitis can arise from bacterial colonization and the formation of resilient microbial communities protected by a glycoprotein mass, as observed in cases of chronic laryngitis [24]. These biofilms pose challenges in treatment due to their resistance to traditional therapies, leading to persistent infections and inflammation in the larynx. Understanding the role of biofilms in LPR and laryngitis is crucial for developing targeted treatment strategies that address the specific challenges these microbial communities pose. Further research into the mechanisms of biofilm formation in the larynx and pharynx is essential for improving diagnostic methods and successfully developing effective therapies to manage biofilm-related laryngopharyngeal conditions.

Biofilms and Tonsillitis

Biofilms play a significant role in chronic tonsillitis, contributing to the persistence and recurrence of infections. These slimy layers of bacteria protect microbes from the host's immune responses and antibiotics, rendering them difficult to eradicate [25,26]. The presence of biofilms in tonsil tissues has been correlated with chronic and recurrent tonsillitis, obstructive sleep apnea, and tonsillar hypertrophy [27]. Research indicates that bacteria-forming biofilms in the tonsils exhibit more excellent antibiotic resistance, posing treatment challenges and leading to chronic inflammation [26]. Understanding the role of biofilms in tonsillitis is paramount for developing effective treatment strategies. Biofilms are regarded as virulence markers and are associated with the chronicity of tonsillar infections, often necessitating tonsillectomy when antibiotic therapy proves ineffective [26]. Accurate visualization and identification of biofilms within tissue sections are essential for precise diagnosis and targeted treatment approaches to address biofilm-related tonsillitis effectively.

Diagnosis of biofilms in otorhinolaryngology

Current Diagnostic Techniques and Challenges

Imaging modalities: Contemporary diagnostic techniques in medical imaging modalities encompass a spectrum of advanced technologies, such as X-rays, computed tomography (CT) scans, magnetic resonance imaging (MRI), positron emission tomography (PET), and ultrasound. These modalities play a pivotal role in diagnosing a myriad of medical conditions, including myocardial diseases, cancer, neurological disorders, congenital heart disease, and abdominal illnesses [28]. Each imaging modality presents unique advantages and challenges in clinical practice. X-rays are extensively utilized for their accessibility and capacity to detect conditions such as pneumonia by visualizing areas of heightened lung density caused by fluid or inflammation [29]. CT scans provide detailed images and are instrumental in diagnosing complex conditions. MRI offers high-resolution images without radiation exposure, rendering it suitable for soft tissue evaluation. PET scans detect tissue metabolic activity effectively, facilitating cancer diagnosis and treatment monitoring [28]. Challenges in medical imaging modalities encompass managing large-scale datasets efficiently, ensuring reliable data interrogation from multiple sources, and optimizing deep learning models for precise diagnoses across diverse populations [30]. Incorporating machine learning techniques such as convolutional neural networks (CNNs) has significantly enhanced the speed and accuracy of diagnoses by automatically classifying imaging data based on computed parameters [29]. Additionally, emerging technologies such as thermography and electrical impedance tomography (EIT) provide non-invasive and radiation-free alternatives for early detection and risk assessment in conditions such as breast cancer [31]. While these innovative modalities offer promising applications, they also encounter specificity, resolution, and regulatory acceptance challenges.

Microbiological cultures: Current diagnostic techniques for bacterial infections encompass a range of microbiological cultures and advanced technologies to identify pathogens and determine antibiotic susceptibility precisely. Traditional methods, such as bacterial cultures with antibiotic sensitivity testing, remain the gold standard for diagnosing bacterial infections [32]. These cultures entail techniques such as Gram staining to identify bacterial morphology and behavior, coagulase and catalase tests to differentiate bacterial species, and blood tests such as full blood count, C-reactive protein, and procalcitonin to evaluate infection severity [32]. Moreover, emerging technologies such as loop-mediated isothermal amplification (LAMP) and DNA microarrays are revolutionizing bacterial detection by providing rapid and accurate results [33]. LAMP assays have been developed for detecting specific bacteria such as Klebsiella pneumoniae, offering swift diagnostic solutions [33]. Additionally, DNA microarrays enable the detection of AMR genes, aiding in understanding bacterial resistance patterns [33]. Challenges in microbiological cultures encompass the limitations of traditional culture-based methods. These methods often fail to detect anaerobic and fungal species, overlook microbial species in biofilms, and inadequately identify AMR potential [34]. Viable but nonculturable (VBNC) pathogens, specimen degradation, systemic antibiotic exposure, and the absence of anaerobe and fungal detection pose significant challenges to accurate diagnosis using culture-based techniques [34]. To address these challenges, advanced diagnostic technologies such as MicroGenDX qPCR+NGS are increasingly utilized to furnish more accurate and actionable microbial infection profiles in chronic infections [34]. This approach, leveraging 16S DNA sequencing, offers a comprehensive and rapid alternative to traditional culture methods, heightening the precision of bacterial identification and treatment selection in clinical settings [34].

Biomarkers and molecular techniques: Current diagnostic techniques in otorhinolaryngology, mainly focusing on biomarkers and molecular techniques, are pivotal in identifying biofilms and associated infections. Biomarkers serve as crucial indicators aiding in the early detection and monitoring of diseases. Within the realm of otorhinolaryngology, biomarkers offer valuable insights into the presence of biofilms and their impact on conditions such as CRS and chronic OM [35,36]. These biomarkers encompass specific proteins, genetic markers, or other molecular signatures indicating the presence of biofilm-related infections. Molecular techniques, such as next-generation sequencing (NGS), have emerged as potent tools for diagnosing biofilms in otorhinolaryngology. NGS accurately identifies the diverse microbial communities within biofilms, providing a comprehensive view of the pathogens involved and their resistance profiles [35]. This advanced molecular approach proves particularly invaluable in cases where traditional culture methods may falter in detecting biofilm bacteria due to their complex structure and resistance mechanisms. Challenges in diagnosing biofilms in ENT include the limitations of conventional culture-based methods in accurately detecting biofilm bacteria and their resistance to antimicrobials. The heterogeneous nature of biofilms, characterized by multiple gradients of anoxic and acidic zones, presents challenges for standard culture techniques, underscoring the necessity for more sophisticated diagnostic tools such as NGS [35]. Additionally, the intrinsic resistance of biofilms to antimicrobial agents further complicates treatment strategies, underscoring the importance of accurate and timely diagnosis employing biomarkers and molecular techniques.

Emerging Technologies and Potential Future Diagnostics

**Figure 2:**
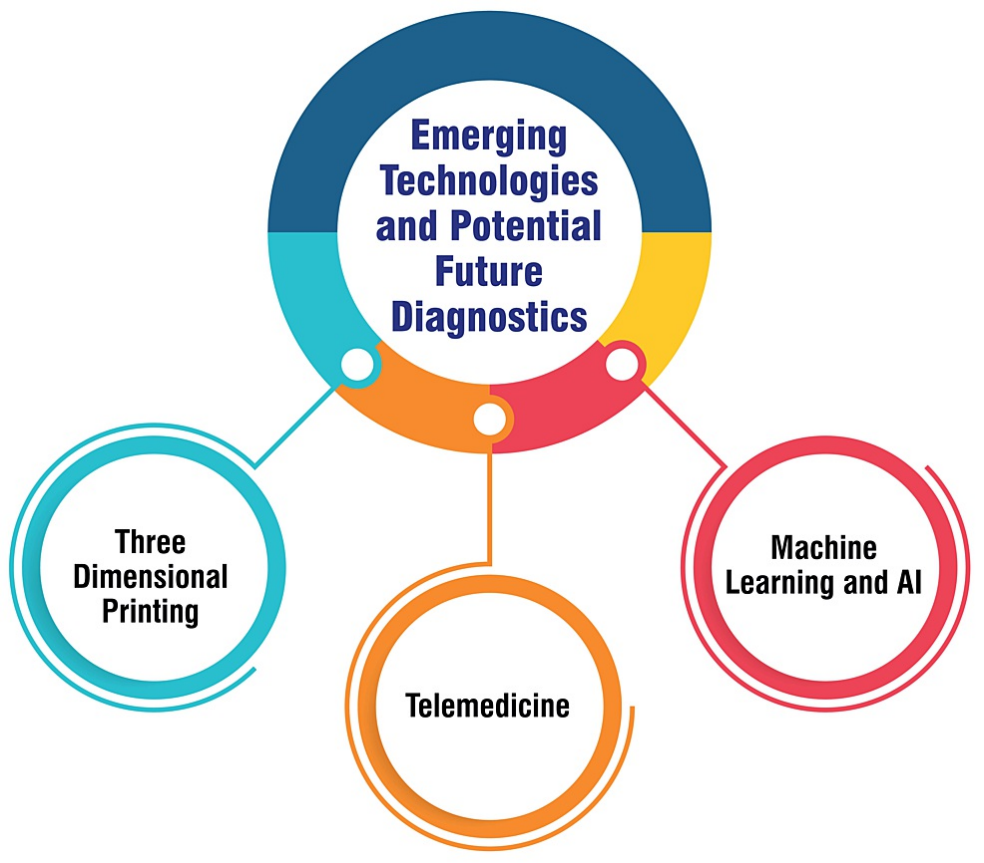
Emerging technologies and potential future diagnostics Corresponding author Dr. Ayushi Ghosh Moulic created this figure.

Machine learning algorithms are currently being employed to analyze video data, aiding in labeling anatomical structures and diagnosing conditions such as OM [37]. Using artificial intelligence (AI) technology, computers can execute tasks that traditionally necessitate human intelligence, augmenting diagnostic accuracy and potentially revolutionizing medical practices within otolaryngology [37]. Active research endeavors are underway to explore the integration of AI into diagnostic systems to enhance patient care. Telemedicine has experienced a significant surge in adoption, particularly amidst the COVID-19 pandemic, offering remote patient care through technological means [37]. Efforts are underway to develop platforms capable of providing objective information, such as audiograms and video otoscopy, thereby broadening the scope of telemedicine in otolaryngologic examinations [38]. Furthermore, smartphones are pivotal in telemedicine applications, facilitating tasks such as hearing loss screening and voice analysis. Three-dimensional printing technology is revolutionizing personalized patient care by enabling the fabrication of intraoperative implants, surgical guides, and anatomical models for training purposes [39]. This innovative technology enhances surgical precision and contributes to residency training in procedures such as peritonsillar abscess drainage and mastoidectomy [39]. Emerging technologies and potential future diagnostics are shown in Figure 2.

Treatment strategies for biofilms in otorhinolaryngology

Conventional Therapies and Limitations

Antibiotics: The utilization of conventional therapies, particularly antibiotics, in addressing biofilm-related infections presents challenges due to the emergence of biofilm resistance. Biofilms demonstrate resistance to antimicrobial agents, including antibiotics, complicating treatment efficacy [40]. This resistance manifests through various mechanisms, such as antibacterial and multidrug resistance, biofilm maturation, dispersal, detachment, and the development of drug resistance within biofilms [40]. Globally, antibiotic resistance is a critical concern, undermining the effectiveness of conventional therapies against biofilm infections. The misuse and overuse of antibiotics significantly contribute to this issue, diminishing biofilms' susceptibility to these medications and exacerbating the problem of drug-resistant infections [40]. Furthermore, instances of self-medication with antibiotics among patients in otorhinolaryngological settings can further exacerbate antibiotic resistance, posing risks to public health by fostering the development of resistant bacterial strains [41]. Given these challenges, it becomes imperative to explore alternative treatment strategies such as nanotechnology-based therapeutics, quorum sensing inhibitors, and surface modifications, alongside judicious antibiotic use, to effectively address biofilm infections while mitigating the risks associated with antibiotic resistance within the context of otorhinolaryngology.

Antimicrobial agents: Conventional antimicrobial agents encounter significant limitations, necessitating exploring alternative therapeutic approaches to combat these challenges. Challenges associated with conventional antibiotics include AMR, ineffectiveness against certain endospores and viruses, and the slow pace of discovering new drugs to counter rapidly growing AMR [42,43]. These hurdles underscore the urgent need for innovative strategies to surmount limitations and enhance the effectiveness of antimicrobial therapies. Consequently, various alternative therapeutic approaches have been proposed to address conventional antibiotics' shortcomings, including new antibiotics combined with β-lactamase inhibitors, phage therapy, antimicrobial peptides, nanoparticles, and antisense antimicrobial therapeutics [42]. Additionally, bacteriophages, antimicrobial peptides, essential oils, and host-oriented therapies demonstrate promise in combating multidrug-resistant organisms and enhancing treatment efficacy [42]. The rise of multidrug-resistant bacteria poses a significant global health threat, prompting a shift towards novel therapeutic strategies that effectively combat infectious diseases while minimizing AMR development. By exploring alternative approaches and integrating different therapies, clinicians can potentially overcome conventional antimicrobial agents' limitations and enhance patient outcomes amidst evolving microbial challenges.

Surgical intervention: Surgical intervention is often deemed more effective than non-surgical treatments for specific conditions such as carpal tunnel syndrome (CTS). Surgical release of the carpal tunnel is recognized as effective, particularly for patients who do not attain relief with conservative approaches such as splinting and steroids. While conservative management may offer relief in certain instances, surgical intervention becomes necessary for definitive treatment, especially when conservative methods prove insufficient [44]. In cancer treatment, traditional therapies such as surgical tumor resection followed by radiotherapy are commonly prescribed. However, these conventional methods possess limitations, including potential harm to healthy cells, chemotherapy resistance, and adverse side effects. Advanced and innovative cancer therapies, such as hormone therapy, immunotherapy, and stem cell therapies, present promising alternatives to mitigate conventional treatment drawbacks. Stem cell therapy, for instance, is being investigated as a safe and effective cancer treatment option, albeit still in the experimental clinical trial phase [45]. Additionally, the comparison between minimally invasive surgery (MIS) and conventional surgery underscores the benefits and considerations of each approach. While MIS techniques offer advantages such as reduced pain, improved recovery, and smaller incisions during hospital stays, they may only sometimes yield long-term benefits akin to conventional methods. The discourse surrounding MIS techniques underscores the significance of meticulously evaluating different surgical approaches' pros and cons to ascertain the best treatment options based on scientific evidence and patient needs [46].

Alternative and Adjunctive Therapies

Biofilm disruptors: Combining natural medicine with conventional therapies such as antibiotics, bacteriophages, and quorum-sensing inhibitors holds promise in managing biofilms. This approach underscores the potential benefits of integrating natural remedies with established treatments to bolster efficacy against biofilms [47]. Combining biofilm dispersal agents with antibiotics is a promising strategy for simultaneously dispersing and eradicating biofilms. This innovative approach presents a novel means of addressing biofilm-related challenges by leveraging the synergistic effects of different agents [48]. Natural biofilm disruptors such as phosphatidylcholine, butyrate, and herbal remedies offer an alternative method to combat biofilms in the gastrointestinal tract. These natural treatments provide a non-conventional yet effective approach to disrupt and manage biofilms in the gut [49]. Researchers have explored physical methods to dismantle EPS within biofilms, enhancing drug diffusion and efficacy against these microbial communities. This direct approach aims to facilitate the penetration of therapeutic agents into biofilms for more favorable treatment outcomes [50]. The resistance of biofilms to antibiotic chemotherapies upon maturation underscores the importance of exploring alternative therapies and disruptors to overcome the limitations of traditional antibiotic treatments against established biofilms [51].

Phage therapy: Phage therapy, which employs bacteriophage viruses to combat bacterial infections, garners attention as an alternative and adjunctive therapy to antibiotics, particularly amidst escalating AMR. This therapeutic approach entails utilizing naturally occurring phages to infect and lyse bacteria, offering a potential solution to combat multidrug-resistant bacterial infections [52,53]. Research suggests that phage therapy holds promise as an alternative or supplementary treatment to antibiotics, with studies demonstrating encouraging results in addressing various bacterial infections, including those caused by Escherichia coli, Vibrio cholerae, Staphylococcus aureus, and Salmonella spp. [52,54]. Phages can be administered orally, topically, directly into body tissues, or systemically, providing flexibility in treatment delivery methods [53]. Despite the promise of phage therapy in combating bacterial infections, its development is challenged by regulatory categorizations that often equate phages to antibiotics. The intricate regulatory landscape can impact the production and conceptualization of phage therapy, impeding its advancement despite growing support from researchers and healthcare professionals [55].

Immunomodulation: Complementary and alternative therapies, including immunomodulation, are under exploration as adjunctive treatments to conventional therapies for various conditions [56]. Host-directed therapies offer an alternative approach by modulating the immune response to infections, potentially serving as adjunctive treatments for conditions such as tuberculosis [57]. Systemic immunomodulation involves using immunosuppressive drugs such as hydroxychloroquine and mycophenolic acid as treatment approaches [58]. In the context of COVID-19, immunotherapies encompassing convalescent plasma, antibodies, cell-based therapies, and immunomodulatory agents are under investigation as alternative options to bolster immunity and address the global crisis precipitated by the pandemic [59].

Probiotics: A study comparing the effects of probiotics and postbiotics derived from Bifidobacterium adolescentis B8589 in a colitis mouse model revealed beneficial effects from both, with postbiotics exhibiting stronger effects on modulating fecal microbiota beta diversity, composition, and metagenomic potential compared to probiotics [60]. Evidence suggests that probiotic supplementation may decrease the incidence of diarrhea, cough, or dyspnea in COVID-19 patients. Additionally, probiotic supplements may enhance clinical outcomes and mitigate adverse events, potentially serving as beneficial adjunctive therapy for individuals with COVID-19 [61]. Probiotic supplements have been demonstrated to be safe and effective adjunctive therapies in managing various conditions, reducing symptoms, improving patient outcomes, and potentially lowering healthcare resource utilization rates, thereby representing a valuable option for patients with diverse health conditions [61]. Probiotics, as living microorganisms, confer health benefits when consumed in sufficient quantities, aiding in enhancing intestinal microbial homeostasis, modulating immune responses, and preventing various infections and diseases [62].

Future Directions in Treatment Development

By 2050, significant evolution is anticipated in clinical trials and drug development. The projected increase in the role of data scientists in clinical trials is set to coincide with the emergence of new technologies and the introduction of a novel three-phase registration model for therapies. This model is envisioned to encompass quality evaluation, safety assessment, and tailored administration options, potentially streamlining drug development processes to be more efficient and patient-centered [63]. Future therapies for cystic fibrosis are exploring a spectrum of approaches aimed at addressing the condition comprehensively. These approaches include antibiotic adjuvants, strategies targeting biofilms and bacteriophages, and treatments focusing on airway surface rehydration and mucus viscosity reduction. Developing new agents is essential to combat common and challenging pathogens, such as non-tuberculous mycobacterium, and to cater to patients who may not benefit from CFTR modulators [64].

**Figure 3:**
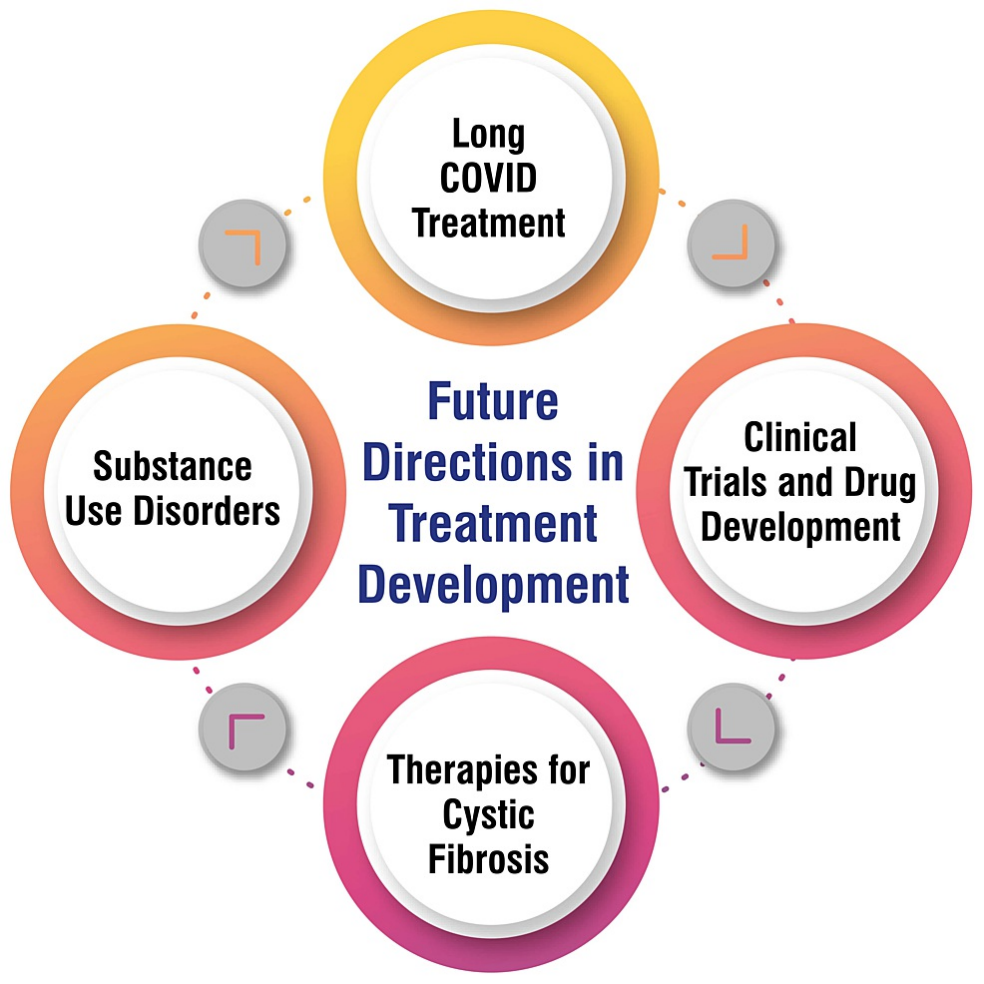
Future directions in treatment development Corresponding author Dr. Ayushi Ghosh Moulic created this figure.

The treatment landscape for substance use disorders is poised for innovation, recognizing the persistent challenges in managing conditions such as alcohol use disorder. Despite the availability of existing interventions, high rates of relapse persist. There is a clear imperative for novel strategies to effectively address substance use disorders, underscoring a growing emphasis on enhancing treatment outcomes in this domain [65]. Future directions in long COVID treatment necessitate a multidisciplinary, holistic approach to enhance patients' quality of life. Various therapies, including physical therapy, medications, and rehabilitation interventions, are being deployed to tackle the diverse symptoms and challenges associated with long COVID. Ongoing research endeavors aim to develop interventions that effectively target the multiple pathways involved in developing long-term COVID [66]. Future directions in treatment development are shown in Figure 3.

Clinical implications and challenges

Impact of Biofilm-Associated Infections on Patient Outcomes

As the findings show, the impact of biofilm-associated infections on patient outcomes is profound. Biofilms present on medical devices such as catheters and implants can precipitate severe complications, prolonged hospital stays, increased healthcare costs, and resistance to antibiotics. These infections pose considerable challenges in treatment owing to the protective shield conferred by biofilms, enabling pathogens to evade both the immune system and antimicrobial agents [67,68]. Studies suggest that patients harboring vital biofilm-producing bacteria face heightened risks of recurrent infections, underscoring the pivotal role of biofilms in fostering persistent infections. While clinical outcomes for patients with biofilm-forming isolates were comparable to those with non-biofilm-forming isolates, mortality rates during the initial infection were found to be higher in patients carrying biofilm-producing bacteria [69]. Discerning the attributes of biofilm-forming bacteria, such as methicillin-resistant Staphylococcus aureus (MRSA) and Pseudomonas aeruginosa, is imperative for identifying individuals at elevated risk of biofilm-related infections. Furthermore, exploring innovative treatment avenues such as antimicrobial coatings for medical devices and alternative therapies such as antimicrobial peptides and bacteriophages holds promise in effectively combating biofilm-associated infections [69,70].

Challenges in Clinical Management and Prevention Strategies

Clinical management and prevention strategies for biofilm infections present notable challenges, particularly in light of the heightened antibiotic resistance observed in bacterial communities within biofilms. This resistance significantly complicates the treatment of biofilm-associated infections, posing a substantial obstacle to successful infection management [51,70]. Various prevention and treatment strategies have been proposed in response to these challenges. These strategies encompass the development of innovative antibiofilm biomaterials, synergistically acting antimicrobial compounds, or a combination thereof. A fundamental aspect of implementing effective preventive and control measures involves a comprehensive understanding of biofilm development mechanisms. Antibiofilm strategies should be centered around several key objectives, including inhibiting microbial adhesion to surfaces, disrupting signal molecules that regulate biofilm development, and disassembling the biofilm matrix [70]. By targeting these critical aspects of biofilm formation, clinicians and researchers can develop more effective approaches to prevent and manage biofilm-associated infections, ultimately improving patient outcomes.

## Conclusions

In conclusion, this review highlights the significant role of biofilms in otorhinolaryngology, particularly in chronic and recurrent infections affecting the ENT. As complex microbial communities with enhanced resistance to antimicrobial agents and host defenses, biofilms pose considerable challenges in diagnosis and treatment. Current diagnostic techniques have limitations, underscoring the need to develop more sensitive and specific tests. Treatment strategies involve a multidisciplinary approach, including antibiotics, antimicrobial agents, surgical intervention, and emerging therapies such as biofilm disruptors and phage therapy. Moreover, the potential of personalized medicine approaches in tailoring treatment strategies for individual patients holds promise. Future research should focus on elucidating the mechanisms of biofilm formation and persistence, developing novel diagnostic techniques, exploring alternative therapies, and integrating personalized medicine into clinical practice. Collaboration between researchers, clinicians, and industry partners will be crucial in translating these advancements into clinically effective interventions, ultimately improving patient outcomes in diagnosing and treating biofilm-related ENT disorders.
